# Terbium ion as RNA tag for slide-free pathology with deep-ultraviolet excitation fluorescence

**DOI:** 10.1038/s41598-019-47353-8

**Published:** 2019-07-24

**Authors:** Yasuaki Kumamoto, Tatsuya Matsumoto, Hideo Tanaka, Tetsuro Takamatsu

**Affiliations:** 1 Department of Pathology and Cell Regulation, Graduate School of Medical Sciences, Kyoto Prefectural University of Medicine, 465 Kajiicho, Kawaramachi-Hirokoji, Kamigyo-ku, Kyoto, 602-8566 Japan; 2 Division of Digestive Surgery, Graduate School of Medical Sciences, Kyoto Prefectural University of Medicine, 465 Kajiicho, Kawaramachi-Hirokoji, Kamigyo-ku, Kyoto, 602-8566 Japan; 3 Department of Medical Photonics, Kyoto Prefectural University of Medicine, 465 Kajiicho, Kawaramachi-Hirokoji, Kamigyo-ku, Kyoto, 602-8566 Japan

**Keywords:** Wide-field fluorescence microscopy, Biomedical engineering

## Abstract

Deep-ultraviolet excitation fluorescence microscopy has enabled molecular imaging having an optical sectioning capability with a wide-field configuration and its usefulness for slide-free pathology has been shown in recent years. Here, we report usefulness of terbium ions as RNA-specific labeling probes for slide-free pathology with deep-ultraviolet excitation fluorescence. On excitation in the wavelength range of 250–300 nm, terbium ions emitted fluorescence after entering cells. Bright fluorescence was observed at nucleoli and cytoplasm while fluorescence became weak after RNA decomposition by ribonuclease prior to staining. It was also found that the fluorescence intensity at nucleoplasm increased with temperature during staining and that this temperature-dependent behavior resembled temperature-dependent hypochromicity of DNA due to melting. These findings indicated that terbium ions stained single-stranded nucleic acid more efficiently than double-stranded nucleic acid. We further combined terbium ions and DNA-specific dyes for dual-color imaging. In the obtained image, nucleolus, nucleoplasm, and cytoplasm were distinguished. We demonstrated the usefulness of dual-color imaging for rapid diagnosis of surgical specimen by showing optical sectioning of unsliced tissues. The present findings can enhance deep-ultraviolet excitation fluorescence microscopy and consequently expand the potential of fluorescence microscopy in life sciences.

## Introduction

Fluorescence microscopes are invaluable tools in the life sciences. They have been widely used by life scientists for analyzing distributions of molecules and cells of interest, as well as cellular and subcellular structures. Recently, advanced techniques, such as super resolution imaging^1–3^, three-dimensional imaging^4,5^, and high-speed imaging^6,7^, have been increasing the importance of fluorescence microscopy in life sciences. In such fluorescence microscopes, visible light (400–700 nm in wavelength, λ) and longer-wavelength ultraviolet (UV) A light (λ = 360–400 nm) are used in general for exciting labeling probes. Though, recent progress in optical technologies and labeling probes drove fluorescence imaging using other wavelengths. As near infrared (NIR) light is barely interactive with biological tissue, NIR excitation fluorescence is studied for deep-tissue molecular imaging and analysis, and a variety of labeling probes for NIR excitation have been developed^8^. In contrast, deep-UV (DUV) light is highly interactive with biological tissue. This property leads to intrinsic small penetration depth of DUV light into a tissue^9^, allowing rapid superficial optical sectioning of an unsliced tissue with a conventional wide-field optical microscope^10,11^, which is applicable to slide-free pathology and rapid diagnosis of a surgical specimen^12^. However, the usefulness of a DUV excitation fluorescence microscope is limited by lack of choices of labeling dyes. No study has been dedicated to establishing a labeling probe for DUV excitation fluorescence microscopy, but just a portion of the fluorescence dyes generally used for visible light excitation (e.g., eosin, Rhodamine, Hoechst, and propidium iodide (PI)) has been diverted^11–15^. Furthermore, such fluorescence dyes are nonspecific to targeted molecules or organelles except for DNA-specific staining dyes, and thereby the capability of DUV excitation fluorescence imaging is limited to distinguishing DNA from others molecules. Development or exploration of specific fluorescence probes can enhance the potential of DUV excitation fluorescence microscopy.

Here, we report the usefulness of terbium ions (Tb^3+^) for visualizing nucleolus in fluorescence microscopy at DUV excitation. As Tb^3+^ enters cells or tissues, it emits bright fluorescence at DUV excitation. The intensity is higher at nucleoli and cytoplasm than at nucleoplasm as Tb^3+^ is specific to RNA. By staining a cell with a mixture of Tb^3+^ and commercial DNA-specific staining dyes, a multicolor fluorescence image where nucleoplasm, nucleoli, and cytoplasm are distinguishable is acquired at DUV excitation. As this multiplex staining does not require any time-consuming and complex procedure, DUV excitation multicolor imaging is useful as an alternative to conventional staining in histopathology for rapid structural imaging. This approach is applicable to rapid diagnosis of a surgical specimen. We expect that the present study enhancing DUV excitation fluorescence microscopy can expand the potential of fluorescence microscopy by enabling biomedical applications that cannot be implemented with visible and NIR excitation.

## Results

**Figure 1:**
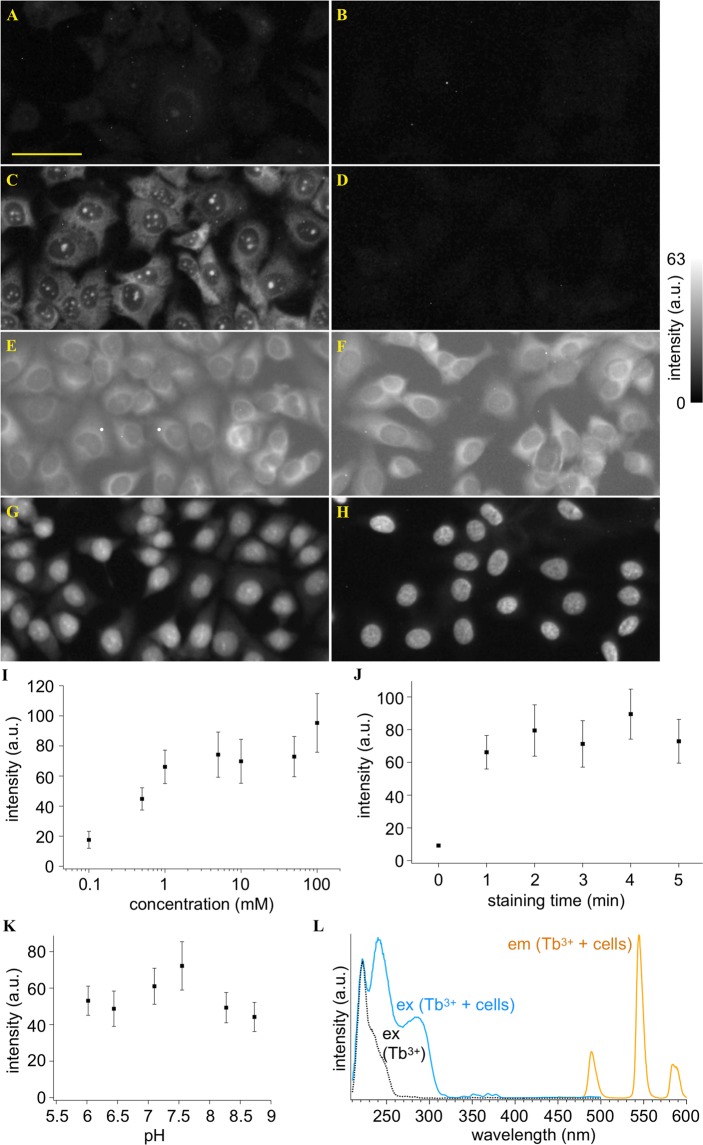
DUV-excitation fluorescence of MCF-7 cells stained with and without Tb^3+^. (**A**–**G**) Fluorescence images of the cells treated with (**A**) a Tb^3+^ solution of H_2_O, (**B**) an H_2_O solution without Tb^3+^, (**C**) a Tb^3+^ solution of D_2_O, and (**D**) a D_2_O solution without Tb^3+^. For comparison, fluorescence images of the cells stained with (**E**) Rhodamine B, (**F**) eosin Y, (**G**) PI, and (**H**) Hoechst 33342 are also shown. Scale bar corresponds to 50 µm. All the images were converted to 8-bits grayscale images from the RGB images measured with an RGB camera. Maximal values of brightness in (**D**–**H**) were adjusted so that difference in the fluorescence intensity distributions could be easily found. Brightness in (**A**–**C**) were adjusted to be the same as that in (**D**) so that the remarkably high fluorescence intensity of Tb^3+^ in D_2_O could be recognized. (**I**–**K**) Dependencies of fluorescence intensity on (**I**) Tb^3+^ concentration, (**J**) treatment period, and (**K**) solution pH. Squares and error bars indicate means and standard deviations of fluorescence intensity for 100 cells under each condition. (**L**) Excitation and emission spectra of the cells immersed in the 10 mM Tb^3+^ solution of D_2_O, as well as an excitation spectrum of the 10 mM Tb^3+^ solution of D_2_O. The excitation wavelength was 285 nm. The emission wavelength detected for measuring the excitation spectra was adjusted to 546 nm. The Tb^3+^ concentration, treatment period, and solution pH were 50 mM, 5 min, and 7, respectively, unless noted.

MCF-7 cells were immersed in a Tb^3+^-containing buffer solution and a standard buffer solution, respectively. Results are shown in Fig. 1A,B. Only the cells immersed in the Tb^3+^ solution showed green fluorescence at DUV (λ = 285 nm) excitation. However, the intensity was weak. We changed the solvent of the Tb^3+^ solution from light water (H_2_O) to heavy water (D_2_O). Figure 1C shows a result of DUV excitation fluorescence imaging of the cells immersed in D_2_O containing Tb^3+^. The cells showed bright fluorescence. Such a distinct increase in the fluorescence intensity was not observed for a D_2_O buffer solution without Tb^3+^ (Fig. 1D). Hence, the combination of Tb^3+^ and D_2_O provided the remarkable fluorescence of the cells. In fact, it was previously reported that D_2_O enhanced Tb^3+^ emission due to suppression of non-radiative decay efficiency of Tb^3+^ ^16,17^.

The image given by Tb^3+^ at DUV excitation (Fig. 1C) highlights nucleoli and cytoplasm. We compared the image and those of the cells stained with different staining dyes reported as DUV-excitable dyes. The cells stained with Rhodamine B^11,14^, eosin Y^11,12,14^, PI^11,12,14^, and 2′-(4-ethoxyphenyl)-5-(4-methyl-1-piperazinyl)-2,5′-bi-1H-benzimidazole trihydrochloride (known as Hoechst 33342)^11,14^ are shown in Fig. 1E–H, respectively. These images are different from the Tb^3+^-staining image; Rhodamine and eosin stained only cytoplasm; PI stained the whole of the nucleus with the slightly higher intensity at nucleoli; Hoechst stained nucleoplasm, highlighting chromatins. It is also noticeable that Rhodamine and eosin, non-specific labeling dyes, also stained outside the cells, *i*.*e*., the buffer solution and the substrate. These comparisons ensure that Tb^3+^ provides a unique and vivid image contrast that cannot be provided by the established dyes.

We examined emission properties of the cells stained with Tb^3+^ in D_2_O. Figure 1I shows fluorescence intensity of nucleoli stained with Tb^3+^ at different concentrations. The graph indicates that concentrations of 1 mM and higher provided bright fluorescence from the stained cells. Shown in Fig. 1J are fluorescence intensities of the cells stained for different treatment times. It was found that the treatment time of 1 min was sufficient for staining the cells with Tb^3+^. Besides concentration of and treatment time with Tb^3+^, pH of the solution can affect excitation and emission efficiencies of Tb^3+^ ^18,19^. Figure 1K represents a pH dependence of the fluorescence intensity. The fluorescence intensity was high at pH 7.1 and 7.55, which are close to the physiological pH (~7.4).

We also investigated excitation and emission spectra of the stained cells (Fig. 1L). At λ = 285 nm excitation, three distinct bands at λ = 490, 546, and 585 nm were observed in the fluorescence spectrum, which are attributed to Tb^3+^ emission^20^. The excitation spectrum acquired with detecting the emission at λ = 546 nm showed three remarkable bands in the DUV range at λ = 220, 240, and 285 nm. Among them, the emission at λ = 220 nm is attributed to any or all of Tb^3+^, Tb^3+^-D_2_O, and Tb^3+^-OD conjugates as it was measured also in the emission spectrum of a Tb^3+^ solution (not containing cells). The other two bands at λ = 240 and 285 nm can be attributed to cellular components. It was reported that Tb^3+^ formed conjugates with chromophores to emit fluorescence at excitation of absorption bands of the chromophores^21,22^, and hence the emission at λ = 240 and 285 nm can be attributed to conjugates of Tb^3+^ and cellular components absorbing DUV light.

**Figure 2:**
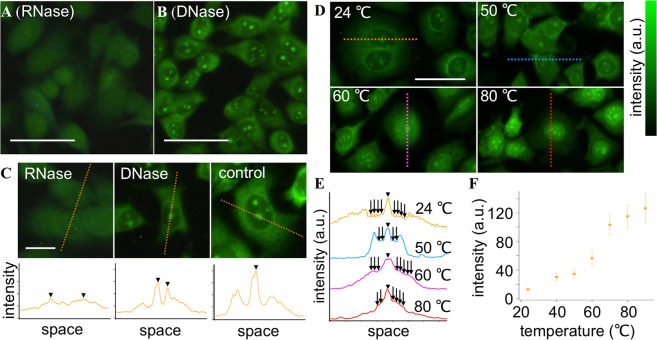
Cytochemical and spectroscopic analyses for revealing Tb^3+^-stained molecules in the cells. (**A**,**B**) DUV-excitation fluorescence images of cells treated with (**A**) RNase and (**B**) DNase and subsequently stained with Tb^3+^. (**C**) Line profiles of the Tb^3+^ fluorescence intensity for RNase-treated, DNase-treated, and non-treated (control) cells. (**D**–**F**) Temperature-dependent Tb^3+^ staining results. Representative fluorescence images of the cells stained with Tb^3+^ at 24, 50, 60, and 80 °C are shown in (**D**). Brightness was adjusted for all the images so that fluorescence distribution can be clearly seen. Line profiles of raw data (meaning the fluorescence images without the brightness adjustment) are shown in (**E**). Arrowheads indicate nucleoli. Arrows indicate nucleus regions. The nucleoplasm intensity was analyzed for 100 cells at each temperature and the results are shown in (**F**). Error bars indicate standard deviations. The excitation wavelength was 285 nm. The Tb^3+^ concentration, treatment period, and solution pH were adjusted to 50 mM, 5 min, and 7, respectively, unless noted. Scale bars in (**A,B,D**) and (**C**) correspond to 20 and 50 µm, respectively.

Because nucleotide and protein are the prominent DUV-absorbers in a cell^23^, we hypothesized that these molecules mainly contribute to the fluorescence from the stained cells at DUV excitation. It was previously reported that RNA^24,25^, DNA^25,26^, and protein^20,25,27^ worked as energy donors for Tb^3+^ as an acceptor to emit fluorescence at DUV excitation. Among these molecules, we focused on RNA because it is condensed at nucleoli as ribosomal RNA (rRNA) and distributed in cytoplasm as rRNA, micro-RNA, and messenger RNA, possibly accounting for the fluorescence intensity distribution of the image shown in Fig. 1C. We used ribonuclease (RNase) to decompose RNA of the cells prior to staining with Tb^3+^. Figure 2A shows a DUV excitation fluorescence image of the cells with the RNase treatment followed by Tb^3+^ staining. Nucleoli were no longer highlighted in the image. This result indicates that RNA conjugated to Tb^3+^ was attributed to the fluorescence from nucleoli. In contrast, decomposition of DNA by deoxyribonuclease (DNase) prior to Tb^3+^ staining (Fig. 2B) hardly changed the fluorescence intensity distribution of the cells in comparison to the cells without nuclease treatment (Fig. 1C). The effects of the nuclease treatments on the Tb^3+^ fluorescence image are also found in line profiles of the fluorescence images (Fig. 2C). These results indicated that Tb^3+^ has selectivity to RNA in staining nucleic acids.

To understand the specificity of Tb^3+^, we considered that most of RNA and DNA in a cell are single-stranded and double-stranded, respectively. Indeed, specificity of the commercial RNA probe which has been widely used in biomedical research^28–30^ is attributed to its specificity to single-stranded nucleic acids. Hence we hypothesized that the specificity could be attributed to the difference of single-stranded and double-stranded structures between RNA and DNA. Considering that a double-stranded nucleic acid is dissociated into single-stranded ones by heating, we analyzed temperature dependencies of the Tb^3+^ staining. Normalized fluorescence intensity images of the heat-treated cells as well as representative line profiles are shown in Fig. 2D,E. At the temperature of 50 °C, nucleoli and cytoplasm were stained well while nucleoplasm was not, like in the non-heated case at 24 °C. This type of distribution no longer appeared when the temperature was increased more; the fluorescence intensity at nucleoplasm rose relatively to that at cytoplasm with temperature increases to 60 and 80 °C. To quantify this temperature-dependent behavior, we analyzed the fluorescence intensities at nucleoplasm of the cells stained at different temperatures. Results are shown in Fig. 2F. The intensity increased instantly between 50 and 70 °C, while it is nearly plateau below 50 °C and above 70 °C. This temperature-dependent behavior resembles hypochromicity of DNA due to dissociation of double strands to single ones^31^. In considering that double-stranded nucleic acids are densely localized in the nucleus as DNA, the temperature-dependent behavior indicates that the intensity increment at nucleoplasm can be attributed to dissociation of the hydrogen bonding in double-stranded nucleic acids. Hence, these results and analyses indicate that single-stranded nucleic acid fluoresces more efficiently in comparison to double-stranded nucleic acid in conjugation with Tb^3+^ under DUV excitation. Indeed, it was shown that base pairing in nucleic acid quenched fluorescence from a conjugate of nucleic acid with Tb^3+^ ^26^, supporting our results indicating the selective labeling of single-stranded nucleic acid, mostly RNA, by Tb^3+^.

**Figure 3:**
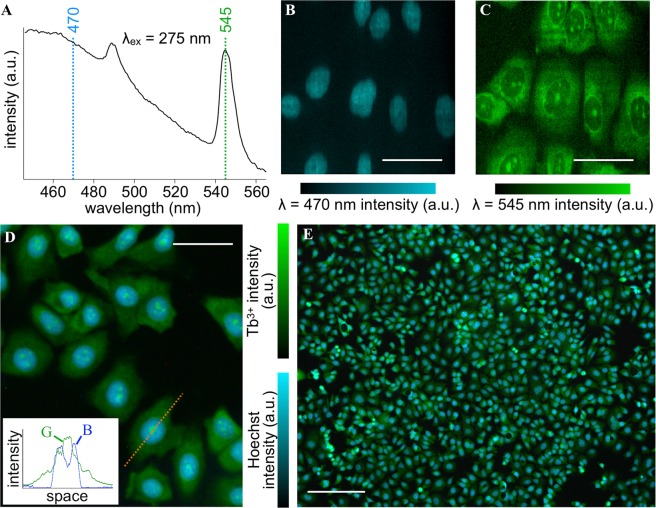
Dual-color fluorescence imaging of the cells stained with Tb^3+^ and Hoechst. (**A**) Representative fluorescence spectra of the cells stained with Hoechst + Tb^3+^. The excitation wavelength was 275 nm. (**B**,**C**) Representative images reconstructed with intensity at (**B**) λ = 470 nm (Hoechst) and (**C**) λ = 545 nm (Tb^3+^), as results of fluorescence hyperspectral imaging. (**D**,**E**) Wide-field imaging of the cells stained with Hoechst + Tb^3+^. The excitation wavelength was 285 nm. A zoom-in and large field-of-view images are shown in (**D**,**E**), respectively. Line profiles are shown in the inset of (**D**). G and B indicate green and blue channels of the color camera, respectively. The Tb^3+^ concentration, treatment period, and solution pH were adjusted to 50 mM, 5 min, and 7, respectively. Scale bars in (**B–D**) and (**E**) correspond to 50 and 200 µm, respectively. The concentration of Hoechst was 10 µg/ml.

As the Tb^3+^ staining is specific to RNA, we considered its combinational use with DNA-specific staining dyes for multicolor imaging. Figure 3A shows representative fluorescence spectra of the cells stained with Tb^3+^ and Hoechst 33342. The Hoechst dye showed a broad fluorescence spectrum in the blue range, while Tb^3+^ showed two sharp bands at λ = 490 and 545 nm^20^. We performed fluorescence hyperspectral imaging of the cells stained with Tb^3+^ and Hoechst, and reconstructed the images with the intensity at λ = 470 and 545 nm (Fig. 3B,C). The Hoechst image presented a typical DNA distribution of a cell, while the Tb^3+^ image showed a similar distribution to the one in Fig. 1C. Similar results were obtained when 4′,6-diamidino-2-phenylindole (known as DAPI) was used instead of Hoechst (Fig. S1). All these results indicate that Tb^3+^ and DNA-specific staining dyes do not interfere with each other for labeling targeted molecules in a cell as well as that they are spectrally separated so that high-contrast multicolor imaging is enabled.

A multicolor fluorescence image can also be acquired with a wide-field microscopic configuration easily and quickly in contrast to the hyperspectral imaging configuration. We used a wide-field fluorescence microscope equipped with an optics enabling oblique illumination with DUV light of a sample. Results of the wide-field multicolor imaging are shown in Fig. 3D. Nucleoplasm was well stained with Hoechst, showing blue fluorescence at DUV excitation, whereas cytoplasm and nucleoli with Tb^3+^ showed green fluorescence. In obtaining these images, we used the dual bandpass filter allowing light of λ = 450–480 and 530–570 nm to be selectively detected. This filter not only rejected the short-wavelength light blurring the image due to chromatic aberration of the optics but also practically widened the dynamic ranges of Hoechst and Tb^3+^ channels by assigning Hoechst fluorescence (λ = 450–480 nm) to the blue channel and Tb^3+^ fluorescence (λ = 545 nm) to the green channel of the RGB camera equipped with the microscope.

The acquisition time for taking a single dual-color image as large as 1.2 × 1.0 mm^2^ in field of view with a 10× objective lens (Fig. 3E) was 500 msec or even shorter. Furthermore, the multiplex staining using Tb^3+^ and DNA-specific dyes was simple and quick; we mixed Tb^3+^ and Hoechst in D_2_O prior to staining, immediately applied this mixture to the cells fixed with ethanol, and waited for 5 min or less for reproducible and regular staining. Such simple and quick multicolor imaging using Tb^3+^ and a DNA-specific dye can be useful for practical applications in cellular biology and medicine.

**Figure 4:**
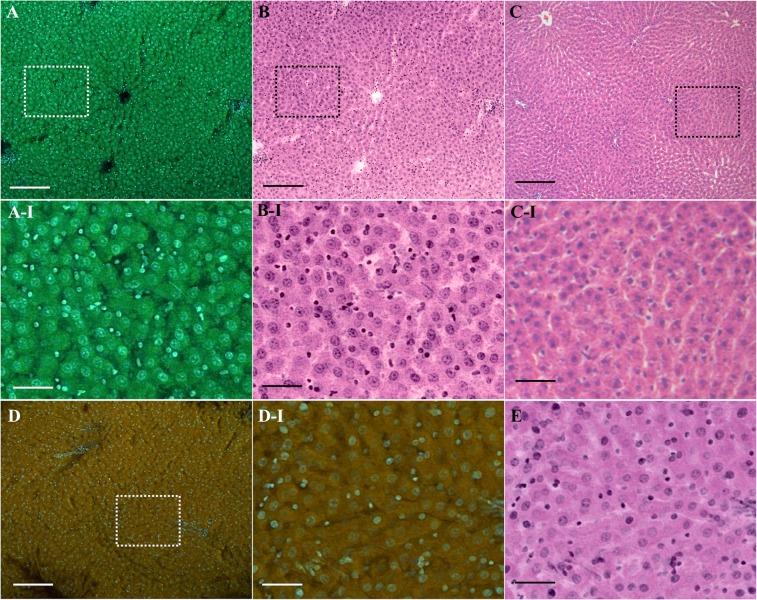
Unsliced tissue imaging with a wide-field DUV-excitation fluorescence microscope. (**A**) An adult rat liver tissue stained with the D_2_O solution containing Tb^3+^ (50 mM) and Hoechst (20 µg/ml). The excitation wavelength, treatment period, and solution pH were adjusted to 285 nm, 3 min, and 7, respectively. The virtual H&E images generated from the fluorescence images shown in (**A**). (**C**) An H&E stain image of an adult rat liver. (**D**) For comparison, the fluorescence image measured with the conventional staining protocol using Hoechst 33342 and Rhodamine B is shown. The regions indicated in (**A**–**D**) by dotted squares are magnified in (**A-I**–**D-I**) respectively. (**E**) The virtual H&E image corresponding to (**D-I**). Scale bars correspond to 200 and 50 µm for the large field-of-view (**A**–**D**) and magnified (**A-I**,**B-I**,**C-I**,**D-I**,**E**) images, respectively. For the fluorescence images shown in (**A**,**D**,**A-I**,**D-I**) unsharp masking was applied. The correspondent original images are shown in Fig. S2. The relatively large cells are hepatocytes, while the small cells seen in the fluorescence and virtual H&E images can be sinusoidal cells such as epithelial cells, Kupffer cells, and stellate cells.

The structural information revealed by the multicolor imaging by Tb^3+^ and Hoechst can be useful for pathology. We explored the potential usefulness of the multicolor imaging for slide-free pathology as one advantage of DUV excitation fluorescence microscopy is the capability of optical sectioning of the sample surface with a wide-field microscope configuration^9–12^. We stained and measured unsliced liver tissue of an adult rat. Results are shown in Fig. 4A. Cells showed bright blue and green fluorescence of Hoechst and Tb^3+^, respectively. The cellular structures such as nuclei, cytoplasm, and nucleoli are visualized by Tb^3+^ and Hoechst, as clearly shown in the magnified image (Fig. 4A-I). We converted the fluorescence images to “virtual hematoxylin and eosin (H&E) images” (Fig. 4B,B-I) with our original protocol (see SI for the algorithm) for comparison with the gold standard histopathological stain, i.e., H&E stain (Fig. 4C,C-I). Despite that the tissue was not paraffin-embedded and thin-sliced, the structural information provided by the fluorescence image was similar to that obtained through the H&E stain image. It is also noticeable that nuclei look larger in the virtual H&E image (Fig. 4B-I) than in the H&E image (Fig. 4C-I). We attributed cause of the difference in size to preprocessing of H&E stain, i.e., formalin fixation and dehydration, as we observed nucleus shrinkage also in a fluorescence image of a formalin-fixed, paraffin-embedded liver slice as well as in an H&E image of a frozen liver slice whereas we did not observe shrinkage in a fluorescence image of a frozen liver slice (Fig. S3). No obvious nucleus shrinkage can be an advantage of the presented slide-free technique over the conventional technique using H&E stain slides.

To clarify uniqueness of the presented staining using Tb^3+^ and Hoechst, we compared the resultant images with the images measured with the conventional fluorescence staining for slide-free histology by DUV excitation fluorescence^11^. Resultant fluorescence and corresponding virtual H&E images are shown in Fig. 4D,E. The virtual H&E images shown in Fig. 4B-I,E look, at a glance, similar to each other, but the intranuclear structures seen in Fig. 4E reside off center and can be chromatins and/or heterochromatins, stained with Hoechst. Additionally, the image contrast is inferior to that provided by Tb^3+^ and Hoechst. For tissues of the other organs such as esophagus and kidney, we observed the same tendencies; the staining using Tb^3+^ and Hoechst provided the higher image contrast and the clearer nucleolus visibility (Fig. S4). With the results, we concluded that the presented staining using Tb^3+^ provided the unique image that could not be provided by the established dyes for slide-free histology.

**Figure 5:**
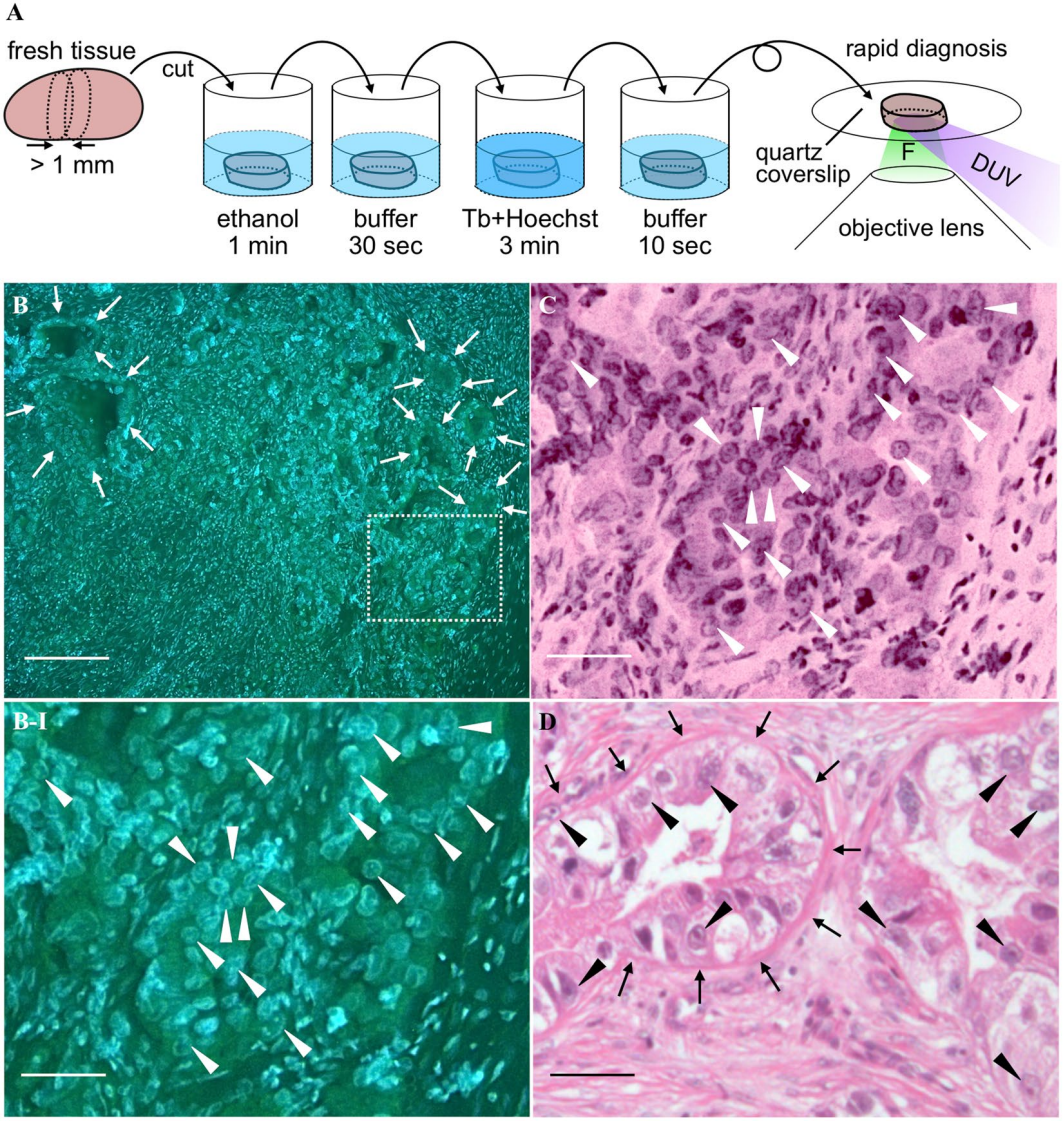
Preclinical demonstration of DUV-excitation fluorescence microscopy with Tb^3+^ and Hoechst for intraoperative rapid diagnosis of lymph node metastasis. (**A**) A schematic view representing the protocol of intraoperative rapid diagnosis of a surgical specimen, drawn by Y.K. Because the lymph node is less contaminated by blood, the tissue is not rinsed before the ethanol treatment, unlike the staining protocol presented in Methods, so that the treatment time is shorten. F: fluorescence. (**B**) A cancer-metastasized lymph node stained with the D_2_O solution containing Tb^3+^ (50 mM) and Hoechst (20 µg/ml). The excitation wavelength, treatment period, and solution pH were adjusted to 285 nm, 3 min, and 7, respectively. The region indicated by the dotted square is magnified in (**B-I**). (**C**) The virtual H&E image corresponding to (**B-I**). (**D**) H&E image of the correspondent lymph node sample. Scale bars correspond to 200 and 50 µm for the large field-of-view (**B**) and magnified **(B-I,C,D)** images, respectively. For the images shown in (**B**,**B-I)** unsharp masking was applied. The correspondent original images are shown in Fig. S2. Arrows indicate boundaries between gland-like structures, which do not exist in non-metastasized lymph nodes, and normal lymph node structures, such as lymphocytes. Arrows indicate the nuclei where nucleoli are found as green-fluorescent particles.

Sample preparation for the unsliced tissue fluorescence imaging was simple and quick. We thereby propose use of the presented staining and imaging for intraoperative rapid diagnosis of surgical specimens. Figure 5A shows the schematic view representing a protocol using the presented technique for rapid diagnosis of a surgical specimen. All the procedures in total takes 5 min approximately. This is much shorter than the standard protocol for intraoperative rapid diagnosis using an H&E stain slide of a thin-sliced frozen tissue (i.e., 20–30 min). We applied this protocol to the fresh lymph node sample resected from a gastric cancer patient. Figure 5B shows the fluorescence image of the lymph node sample. There are relatively small and large cells in the image. The smaller cells are lymphocytes, existing originally in a non-metastasized lymph node, whereas the larger cells can be tumor cells metastasized from the stomach. However, cellular size is not the deterministic factor for identifying tumor cells in a lymph node by pathologists; nucleoli as well as gland-like structures and nuclear atypia are also considered for pathological diagnosis of cancer metastasis. In the fluorescence images, nucleoli are found as green-fluorescent particles in the nuclei indicated by arrowheads (Fig. 5B-I), while the gland-like structures are found at the regions indicated by arrows (Fig. 5B). Additionally, nuclei with low circularities can be identified, thanks to the good image contrast provided by Tb^3+^ and Hoechst. Hence, a pathologist identified the lymph node as positive metastasis and this identification result matched with the result with the conventional H&E stain slide (Fig. 5C). DUV excitation fluorescence microscopy combined with the multiplex staining using Tb^3+^ and Hoechst is a promising tool for accelerating intraoperative rapid diagnosis of surgical specimens.

## Discussion

DUV excitation fluorescence microscopy allows optical sectioning with a wide-field microscopic configuration^9,10^. This modality, known as microscopy with ultraviolet surface excitation, namely MUSE, is promising for intraoperative diagnosis of surgical specimens^11,12,14^, whereas further studies on fluorescent probes are essential for practical applications. The present study reporting the first fluorescent probe designated for DUV excitation can drive exploration and development of DUV-excitable fluorescent probes. Moreover, it is of great significance that we found the nucleolus staining technique that allows combinational use with nucleoplasm-staining dyes for MUSE. Because the nucleus shape, nucleus-cytoplasm size ratio, and nucleolus shape of cells are important morphological factors for cancer diagnosis by pathologists, the present staining methods can enhance the usefulness of MUSE for clinical applications.

Tb^3+^ and Hoechst fluorescing in blue and green reserve the red channel of the RGB camera for another dye. This reserved channel can be used for detecting a red-fluorescent dye. Since the staining with Tb^3+^ and Hoechst could lose the information provided by eosin Y such as whether a cell is eosinophilic or not, eosin B, excitable at DUV light and fluorescing in red, can be a candidate for another dye.

There are other things to discuss in practical applications of MUSE. Although the penetration depth of DUV light into biological tissue is limited, it is no less than 20 µm^12^. This value is larger than the depth of focus of optical microscopy in general (<10 µm) and thus acquired images with a wide-field DUV microscope often include blurred structures existing out of the focal plane. To overcome this limitation, several approaches have been studied in recent years, including water immersion of objective and illumination lenses for large-angle oblique illumination^12^ and three-dimensional deconvolution for extending the depth of focus^15^. Of them, the deconvolution process eliminates image blurring not only due to the penetration depth limitation but also due to roughness of the sample surface, and hence is a powerful tool. However, it requires several images acquired from different planes, making data acquisition time longer. This is not desirable for clinical applications in which a large surgical tissue needs to be diagnosed as rapidly as possible. One key technology shortening data acquisition time can be DUV light-emitting diode (LED). Recent advances in DUV LED are remarkable and the optical power of DUV LED has been increasing. We expect further increases in this optical power.

It is also worth noting the potential usefulness of the Tb^3+^ labeling in cell biology. RNA has become an important target in the life sciences^32,33^. While fluorescence *in situ* hybridization^34^, transfection with fluorescent protein^35^, and molecular beacons^36^ are widely used for measuring specific RNAs, non-specific but ready-to-use RNA probes have also been developed recently^37–41^. Such ready-to-use RNA probes are useful for quick RNA labeling, and Tb^3+^ staining, enabling bright fluorescence imaging of RNA with easy and reproducible staining, can be as well. One limitation of the Tb^3+^ staining in this aspect can be possible influence of other molecules. We found that Tb^3+^ highlighted cytoplasm more vividly than commercial RNA-staining dyes (Fig. S5). Although Tb^3+^ might fairly reflect a massive amount of rRNA existing in cytoplasm, the difference also can imply the possibility of contribution to the Tb^3+^ fluorescence images from other molecules, such as protein; protein is the other prominent DUV-absorber in addition to nucleic acid in cells and tissues. According to the literature, proteins having metal-binding sites^20^ and Ca^2+^-binding sites^27^ can work as energy donors for Tb^3+^ as an acceptor to fluoresce. These proteins can contribute to the Tb^3+^ fluorescence coming from a cell, reducing the accuracy of the Tb^3+^ staining for quantifying RNA contents in cells and tissues. Further studies are necessary for quantifying the influence of proteins on the cell staining by Tb^3+^, although the temperature- and nuclease-dependent characters of Tb^3+^ staining of cells indicate that fluorescence from Tb^3+^ conjugated with single-stranded nucleic acid dominate the fluorescence signals of the cells stained by Tb^3+^.

## Methods

### Cells

MCF-7 cells (EC86012803, European Collection of Authenticated Cell Cultures), numbering 2 × 10^5^, were seeded on a fused silica coverslip with a thickness of 0.13–0.17 mm (SF-S-D12, Fine Plus International) or a glass coverslip (D11130H, Matsunami Glass Industry). Dulbecco’s modified Eagle’s medium (D5546, Sigma Aldrich) supplemented with 10% fetal bovine serum (AC10235246, SH30910.03, Hyclone) and 1% penicillin-streptomycin-glutamine (161–23201, Nacalai Tesque) was used as a culturing medium. The cells were cultured at 37 °C and in 100% relative humidity in the 5% CO_2_ atmosphere for 2 days.

### Cytochemical treatments

For the cytochemical treatments using DNase (2270A, Takara Bio) and RNase (U0505S, Takara Bio), the cultured cells were fixed and permeabilized with 99.5% ethanol. For the RNase treatment, the cells were immersed in Hank’s balanced salt solution (HBSS) containing RNase (1 mg/ml) and 0.2% bovine serum albumin (BSA) for 60 min. For the DNase treatment, the cells were immersed in HBSS containing DNase (10 units/ml), MgCl_2_ (5 mM), and 0.2% BSA for 60 min. The cells during nuclease reaction were shaken in a water bath heated up to 30 and 40 °C for RNase and DNase, respectively. After the reaction, the cells were rinsed with HEPES buffer solution (10 mM HEPES, pH = 7 adjusted with NaOH).

### Animals

Adult male Wistar rats at the age of 8 weeks were purchased from Shimizu Laboratory Supplies. All the experiments were performed with the approval of and in accordance with guidelines from the Animal Research Committee of Kyoto Prefectural University of Medicine (Approval No. M29-559). The liver, kidney, and esophagus tissues taken from the rats were immediately immersed in a 0.9% NaCl aqueous saline solution on ice and excised into a small piece (approximately 5 mm × 5 mm × 3 mm in size).

### Clinical specimens

All clinical experiments were conducted with the approval of the Ethics Committees of Kyoto Prefectural University of Medicine (Approval No. ERB-C-1038-1) as well as in accordance with guidelines from the committees and regional laws related to clinical research. Informed consent was obtained from all participants. The lymph nodes taken from a female patient with gastric cancer in the Hospital of Kyoto Prefectural University of Medicine were fixed with 10% formalin and embedded in solid paraffin blocks as a routine work. After the deparaffinization, the serial sections with the thickness of 4 µm were treated by Gill’s H&E staining protocol (see SI for the details) or the following Tb^3+^ staining.

We also used a fresh lymph node taken from a female patient with gastric cancer in surgery operated on the Hospital of Kyoto Prefectural University of Medicine. The piece of the resected lymph node that was not used for routine diagnostic processes was excised into a small piece (approximately 5 mm × 5 mm × 3 mm in size).

### Staining protocol

After rinsing with HEPES buffer solution (10 mM HEPES, pH = 7 adjusted with NaOH), immersion in 95 or 99.5% ethanol, and rinsing with the HEPES buffer, cells and tissues were immersed in 100% D_2_O HEPES buffer solution containing TbCl_3_ (TBH03XB, Kojundo Chemical Laboratory) at concentrations of 0.1–100 mM for 1–5 min. The cells and tissues were rinsed with 100% D_2_O HEPES buffer solution. For multicolor staining, a 100% D_2_O HEPES buffer solution containing TbCl_3_ and Hoechst 33342 (Dojindo Molecular Technologies) or DAPI (Dojindo Molecular Technologies) at the concentrations of 0.1–100 mM and 10–20 µg/ml, respectively, were used. For temperature-dependent staining experiments, the staining solution containing Tb^3+^ at the concentration of 50 mM was heated to 50, 60, 70, 80, and 90 °C prior to application to the ethanol-treated cells, and the cells were subsequently immersed in the heated solution for 5 min at target temperature.

For the conventional staining procedures using Rhodamine B (Nacalai esque), eosin Y (Merck), PI (Dojindo Molecular Technologies), and Hoechst 33342, a buffer solution containing one of those dyes at the concentration of 100 µg/ml (for Rhodamine and PI), 1 mM (eosin Y), or 2 µg/ml was used. For tissue staining, the concentrations of Hoechst 33342 and Rhodamine B were adjusted to 20 and 100 µg/ml, respectively.

### Fluorescence microscopy

We placed the sample on the sample stage of an inverted microscope (IX71, Olympus) equipped with an objective lens (UPLFLN 10×/0.3, Olympus). The sample surface was illuminated with a DUV beam (30 mW and 5 mm in power and diameter, respectively) emitting from an LED (M285L5, Thorlabs). Fluorescence emitting from the sample was collimated with the objective lens and imaged on a CMOS camera (UI-3180CP-C-HQ Rev. 2, OnSemi). An optical filter (LVX450, Asahi Spectra or FF01-464/547-25, Semrock) was placed at the position between the objective lens and the imaging lens. The schematic view of the microscope is shown in Fig. S6.

Fluorescence hyperspectral images were also measured from samples. Briefly, DUV light emitting from an LED (Daico MFG) was shaped into a line with a mechanical slit with the width of 25 µm. The line beam was projected to the sample through an objective lens (UV 50× A, Nikon Engineering), resulting in the estimated width of 0.5 µm. Fluorescence from the illuminated region was imaged at the entrance slit (50 µm) of a spectrometer (MS3504i, SOL Instruments) equipped with a CCD camera (Newton DU920P Bx-DD, Andor Technology). To acquire a two-dimensional spatial distribution of fluorescence spectra, the sample was scanned by the line-shaped DUV light with the assistance of a motorized stage (TS1L80-015, Nanocontrol). The schematic view of the microscope is shown in Fig. S7.

### Excitation and emission spectral measurement

Excitation and emission spectra of the cell sample were measured with a fluorescence spectrophotometer (F7000, Hitachi High-Technologies). Briefly, MCF-7 cells were detached from the culturing dish with trypsin-EDTA (201-16945, Wako Chemicals), permeabilized and fixed with 95% ethanol, and immersed in a buffer solution containing Tb^3+^ at the concentration of 10 mM. The cell suspension was then put in a quartz cuvette containing a rotating microstirrer, and emission and excitation spectra of the cell suspension was acquired. As a reference, the buffer solution containing Tb^3+^ at the concentration of 10 mM was also measured.

### Intensity analysis of the fluorescence images

The acquired wide-field DUV excitation fluorescence images were analyzed with ImageJ (version 1.48 v) for quantitative analysis of fluorescence intensity. Fluorescence images with RGB channels were split into three to extract the green channel attributed to Tb^3+^ fluorescence. After applying a median filter to the green channel image, we took a maximum value from nucleus for obtaining a representative fluorescence intensity of a nucleolus. The maximum values for 100 cells measured under certain conditions (i.e., staining time, pH, and concentration) were obtained and used for calculating the mean and the standard deviation. To analyze nucleoplasm fluorescence intensity, we took a median value from a nucleus. The median values for 100 cells measured at a certain temperature were obtained and used for calculating the mean and the standard deviation.

### Unsharp masking of the unsliced tissue images

Fluorescence images of the unsliced tissue measured with a wide-field DUV fluorescence microscope are blurred relatively to those of the thin-sliced tissue due the larger penetration depth of DUV light to tissue (~10 µm) than the thickness of the sliced section (3–4 µm). To reduce the blurring, we applied unsharp masking to the raw unsliced tissue images. We used imsharpen function in MATLAB for unsharp masking.

### Color conversion generating virtual H&E images from the fluorescence images

We used MATLAB (R2018a, Mathworks) for converting Tb^3+^-Hoechst fluorescence images to virtual H&E images. The original code used is shown in SI.

## Supplementary information

Supporting Information
