# Supplementary material for: Terbium ion as RNA tag for slide-free pathology with deep-ultraviolet excitation fluorescence
Source: Sci Rep. 2019 Jul 24;9:10745. doi: 10.1038/s41598-019-47353-8 (PMC6656878; doi:10.1038/s41598-019-47353-8)
Supplement: Supplementary file 1 — Supporting Information [file 41598_2019_47353_MOESM1_ESM.pdf]

Supporting Information

Terbium ion as RNA tag for slide-free pathology with deep-ultraviolet excitation fluorescence

Yasuaki Kumamoto,[1,*] Tatsuya Matsumoto,[1,2] Hideo Tanaka,[1] Tetsuro Takamatsu[3]

[1]Department of Pathology and Cell Regulation, Graduate School of Medical Sciences, Kyoto Prefectural University of Medicine, 465 Kajiicho, Kawaramachi-Hirokoji, Kamigyo-ku, Kyoto 602-8566, Japan

[2]Division of Digestive Surgery, Graduate School of Medical Sciences, Kyoto Prefectural University of Medicine, 465 Kajiicho, Kawaramachi-Hirokoji, Kamigyo-ku, Kyoto 602-8566, Japan

[3]Department of Medical Photonics, Kyoto Prefectural University of Medicine, 465 Kajiicho, Kawaramachi-Hirokoji, Kamigyo-ku, Kyoto 602-8566, Japan

Table of Contents

1. Fig. S1: Dual-color fluorescence imaging and spectral measurement using DAPI

2. H&E staining and microscopic observation

3. MATLAB code generating virtual H&E images from the fluorescence images

4. Fig. S2: Unsliced tissue images without unsharp masking

5. Fig. S3: H&E and fluorescence images of FFPE and frozen liver slices

6. Fig. S4: Fluorescence, virtual H&E, and H&E images of kidney and esophagus

7. Fig. S5: Fluorescence images of MCF-7 stained with commercial RNA dyes

8. Fig. S6: A wide-field fluorescence microscope at deep-ultraviolet (DUV) excitation

9. Fig. S7: A fluorescence hyperspectral imaging microscope at DUV excitation

1. Fig. S1: Dual-color fluorescence imaging and spectral measurement using DAPI

Representative fluorescence spectra of MCF-7 cells stained with DAPI+Tb^{3+} (left), and wide-field imaging of the cells stained with DAPI+Tb^{3+} (right) are shown. The excitation wavelengths for spectral measurement and wide-field imaging were 275 and 285 nm, respectively. The Tb^{3+} concentration, treatment period, and solution pH were adjusted to 50 mM, 5 min, and 7, respectively. Scale bar corresponds to 50 μm. The concentration of DAPI was 10 μg/ml.

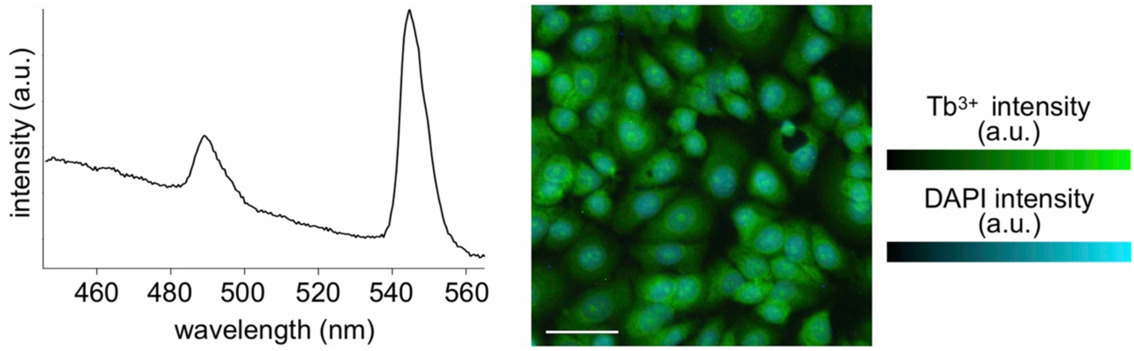

2. H&E staining and microscopic observation

Sliced tissues were treated with the Gill's H&E staining protocol; briefly, the tissues were deparaffinized with xylene and ethanol, stained with Gill's hematoxylin and eosin, dehydrated with ethanol, cleared with xylene, and embedded in a mounting medium. The stained tissue was measured with an inverted microscope (BX51, Olympus) equipped with an objective lense (UPlanSApo 10×, Olympus) and a CCD camera (DFC320, Leica).

3. MATLAB code generating virtual H&E images from the fluorescence images

```
function VirtualHE(FIfilename,var1,var2,var3,var4,var5,var6,var7,var8,var9,var10,var11)
    data = double(imread(FIfilename));
    [NumY,NumX,NumChan] = size(data);
    RG = double(mean(data(:,:,1:2),3));
    B = double(data(:,:,3));
    RGB = double(mean(data,3));
    RGBunb = RGB-min(min(RGB));
    RGBnorm = RGBunb/max(max(RGBunb))*255;
    K = 255-RGBnorm;
    C = B.*power(K,var1)/255^var1;
    M = RG.*power(K,var2)/255^var2;
    Y = zeros(NumY,NumX);
    NewR = (255-C).*power(K,var3)/255^var3;
    ur = max(max(NewR/2))*(1+1/var4);
    lr = max(max(NewR/2))*(1-1/var4);
    NewR(NewR>ur) = ur;
    NewR(NewR<lr) = lr;
    NewR = (NewR-lr)/(ur-lr)*255;
    if var5 == 1
    else
        NewR = NewR+max(max(NewR))*(var5-1);
        NewR(NewR>255) = 255;
        NewR(NewR<0) = 0;
    end
    NewG = (255-M).*power(K,var4)/255^var6;
    ug = max(max(NewG/2))*(1+1/var7);
    lg = max(max(NewG/2))*(1-1/var7);
    NewG(NewG>ug) = ug;
    NewG(NewG<lg) = lg;
    NewG = (NewG-lg)/(ug-lg)*255;
    if var8 == 1
    else
        NewG = NewG+max(max(NewG))*(var8-1);
        NewG(NewG>255) = 255;
        NewG(NewG<0) = 0;
    end
    NewB = (255-Y).*power(K,var9)/255^var9;
    ub = max(max(NewB/2))*(1+1/var10);
    lb = max(max(NewB/2))*(1-1/var10);
    NewB(NewB>ub) = ub;
    NewB(NewB<lb) = lb;
    NewB = (NewB-lb)/(ub-lb)*255;
    if var11 == 1
    else
        NewB = NewB+max(max(NewB))*(var11-1);
        NewB(NewB>255) = 255;
        NewB(NewB<0) = 0;
    end
    [RGB,RGBunb] = max(reshape(NewB.*NewR.*NewG,[NumY*NumX 1]));
    temp = reshape(NewR,[NumY*NumX 1]);
    NewR = NewR/temp(RGBunb)*255;
    temp = reshape(NewG,[NumY*NumX 1]);
    NewG = NewG/temp(RGBunb)*255;
    temp = reshape(NewB,[NumY*NumX 1]);
    NewB = NewB/temp(RGBunb)*255;
    savedata = zeros(NumY,NumX,NumChan);
    savedata = uint8(savedata);
    savedata(:,:,1) = uint8(NewR);
    savedata(:,:,2) = uint8(NewG);
    savedata(:,:,3) = uint8(NewB);
    imwrite(savedata, ‘savefilename’)
end
```

4. Fig. S2: Unsliced tissue images without unsharp masking

Original unsliced tissue images without unsharp masking are shown. The correspondent unsharp-masked images are shown in Figs. 4, S4, and 5. Scale bar corresponds to 200 μm.

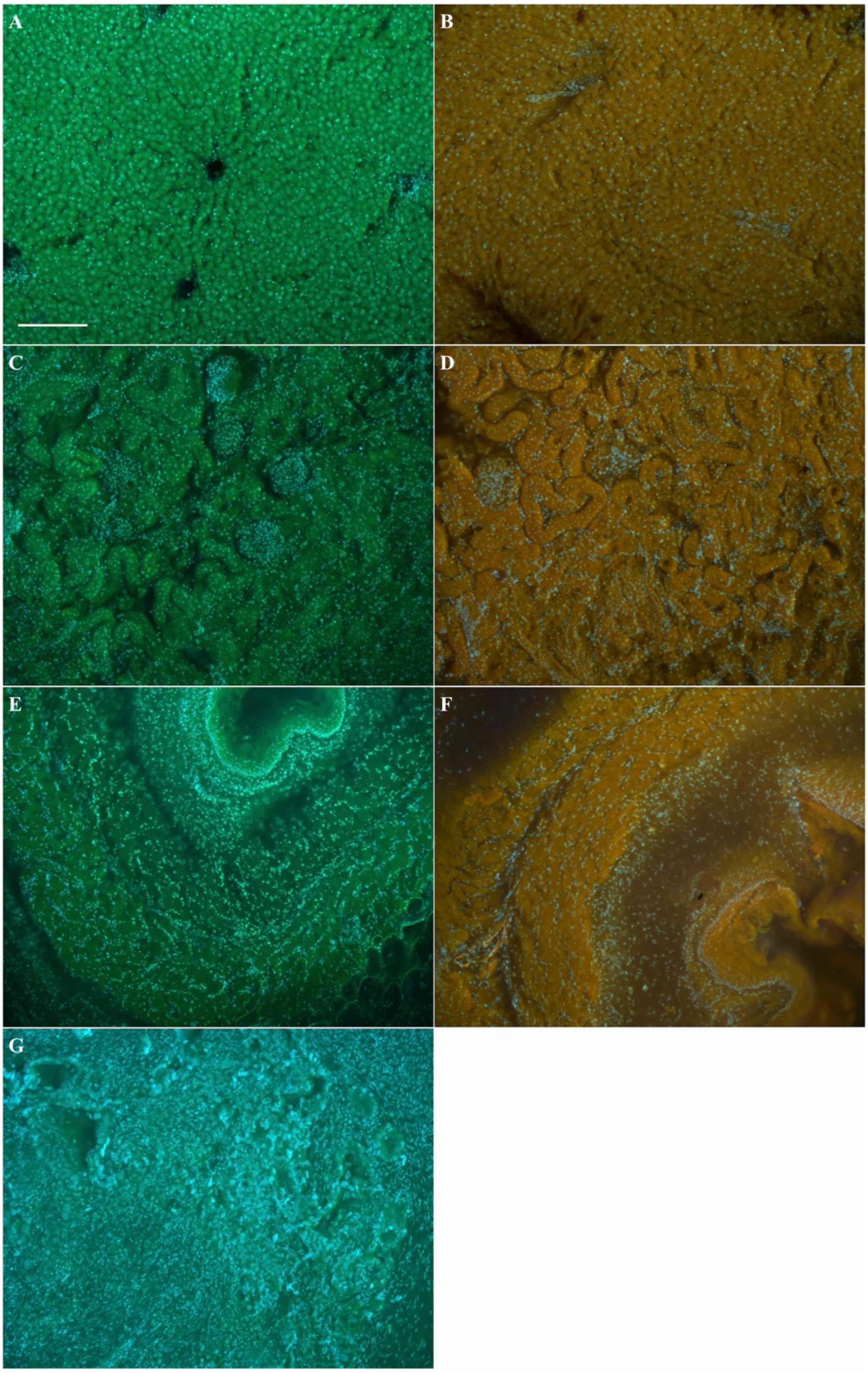

5. Fig. S3: H&E and fluorescence images of FFPE and frozen liver slices

(*A*-*C*) Fluorescence (*A*), virtual H&E (*B*), and H&E (*C*) images of serial slices of an FFPE liver tissue. (*D*-*H*) Fluorescence (*D*, *G*), virtual H&E (*E*), and H&E (*F*, *H*) images of serial slices of a frozen liver tissue. Nuclei look smaller for the fluorescence and H&E images of the FFPE liver slices (*A*-*C*) and the H&E image of the frozen liver slice (*F*) in comparison to the fluorescence image of the frozen liver slice (*D*, *E*). Tissue shrinkage can also be recognized by comparing large field-of-view images (*G*, *H*). Scale bars correspond to 200 and 50 μm for the magnified (*A*-*F*) and large field-of-view (*G, H*) images, respectively.

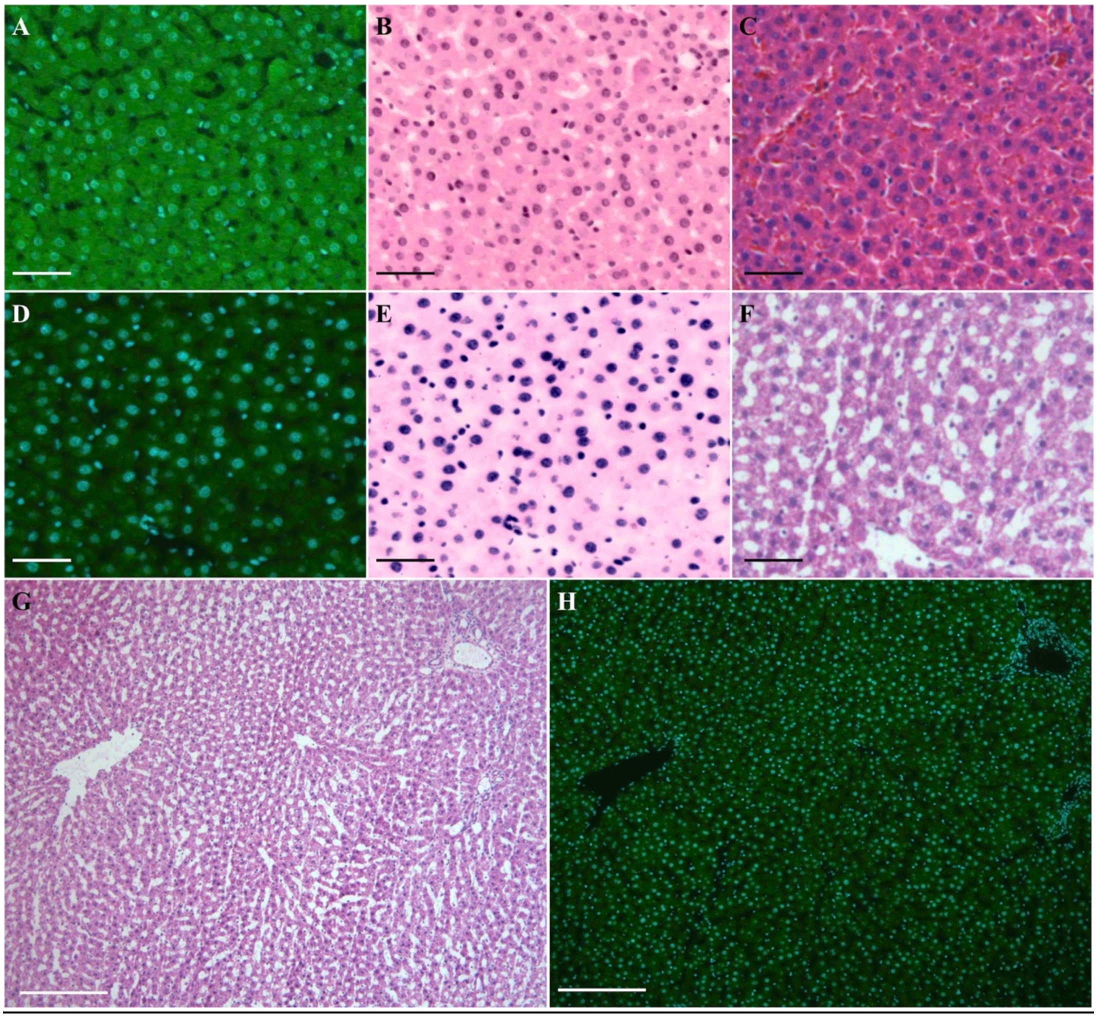

6. Fig. S4: Fluorescence, virtual H&E, and H&E images of kidney and esophagus

(*A*) A fluorescence image of an adult rat kidney tissue stained with Tb^{3+} and Hoechst. (*B*) The virtual H&E image corresponding to *A*. (*C*) An H&E stain image of the adult rat kidney. (*D*) The fluorescence image measured with the conventional staining protocol using Hoechst and Rhodamine. (*A*-I, *B*-I, *C*-I, *D*-I) Magnified views of the regions indicated by dotted squares. (*E*) The virtual H&E image corresponding to *D*-I. (*F*) A fluorescence image of an adult rat esophagus tissue stained with Tb^{3+} and Hoechst. (*G*) The virtual H&E image corresponding to *F*. (*H*) An H&E stain image of the adult rat kidney. (*I*) The fluorescence image measured with the conventional staining protocol using Hoechst and Rhodamine. (*F*-I, *G*-I, *H*-I, *I*-I) Magnified views of the regions indicated by dotted squares. (*J*) The virtual H&E image corresponding to *I*-I. Scale bars correspond to 200 and 50 μm for the large field-of-view (*A*, *B*, *C*, *D*, *F*, *G*, *H*, and *I*) and magnified (*A*-I, *B*-I, *C*-I, *D*-I, *E*, *F*-I, *G*-I, *H*-I, *I*-I, and *J*) images, respectively. For the fluorescence images, unsharp masking was applied. The correspondent original images are shown in Fig. S2. In the kidney images, glomeruli (the circular structures densely containing a number of nuclei) and renal tubules (the curving belt-like structures) are visualized. Nucleoli are found in the cells at renal tubules shown in *A*-I, *B*-I, and *C*-I. In the esophagus images, layer structures can be identified, including mucosal, submucosal, and proper muscular layers. Nucleoli are found in the cells at the mucosal layer shown in *F*-I, *G*-I, and *H*-I.

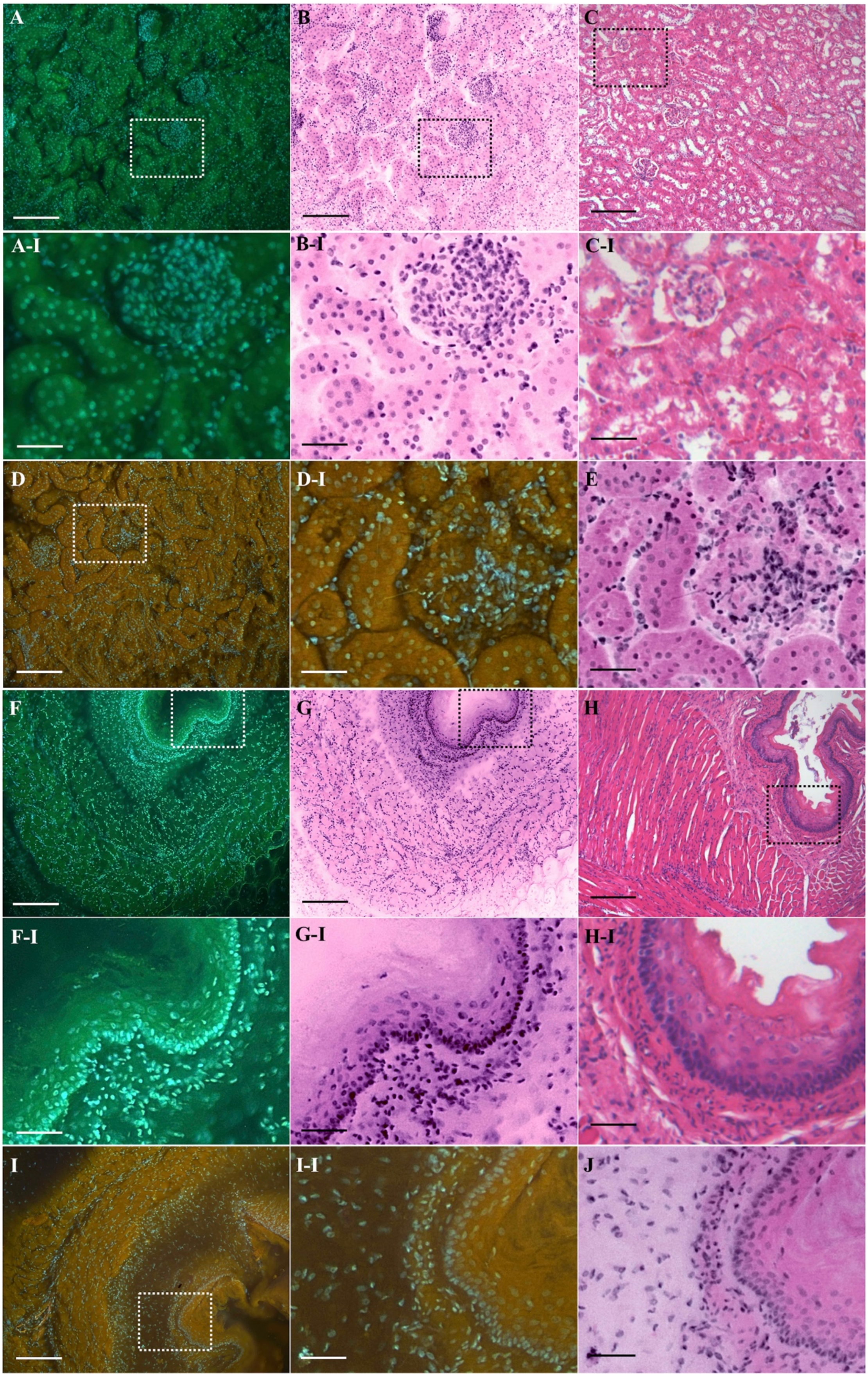
A
B
C
A-I
B-I
C-I
D
D-I
E
F
G
H
F-I
G-I
H-I
I
I-I
J

7. Fig. S5: Fluorescence images of MCF-7 stained with commercial RNA dyes

We stained MCF-7 cells with commercial RNA dyes, i.e., StrandBrite RNA Green (AAT Bioquest) and Syto RNAselect Green Fluorescent Cell Stain (Thermo Fisher). Pretreatment performed prior to the staining, i.e., washing with a buffer solution and immersing in ethanol, was the same as those for Tb^{3+} staining described in Methods section. The concentration of the Syto dye was 1 μM. For the StrandBrite, we adjusted the concentration (1/200 dilution) according to the protocol provided by the maker. The staining time was 30 min. Because these dyes could not be excited at DUV, we used another fluorescence microscope (IX70, Olympus) equipped with a blue light LED (M470L3, Thorlabs) and a suitable filter set (U-MNIBA, Olympus) as well as a monochromatic CMOS camera (DCC1545M, Thorlabs) and an objective lens (UPlanFl 20×/0.5, Olympus) for fluorescence imaging. The resultant images are shown below. (*A*) The cells stained with StrandBrite RNA Green. (*B*) The cells stained with SYTO RNAselect. Scale bar corresponds to 50 μm. Brighter nucleoplasm fluorescence in *B* than in *A* can be explained by non-specific DNA fluorescence of Syto RNAselect. See the following URLs (as of 21/06/2019) for details of non-specific DNA labeling of Syto RNAselect.

https://assets.thermofisher.com/TFS-Assets/LSG/manuals/mp32703.pdf

http://aatbioquest.blogspot.com/2017/03/live-cell-rna-imaging-kit.html

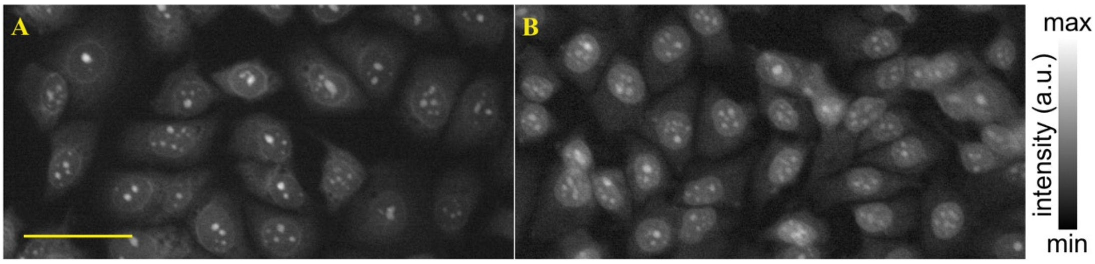

8. Fig. S6: A wide-field fluorescence microscope at DUV excitation

We built a wide-field fluorescence microscope equipped with an optics enabling oblique illumination with DUV light of a sample. A schematic view representing the microscope, drawn by Y.K., is shown. Details are described in Methods.

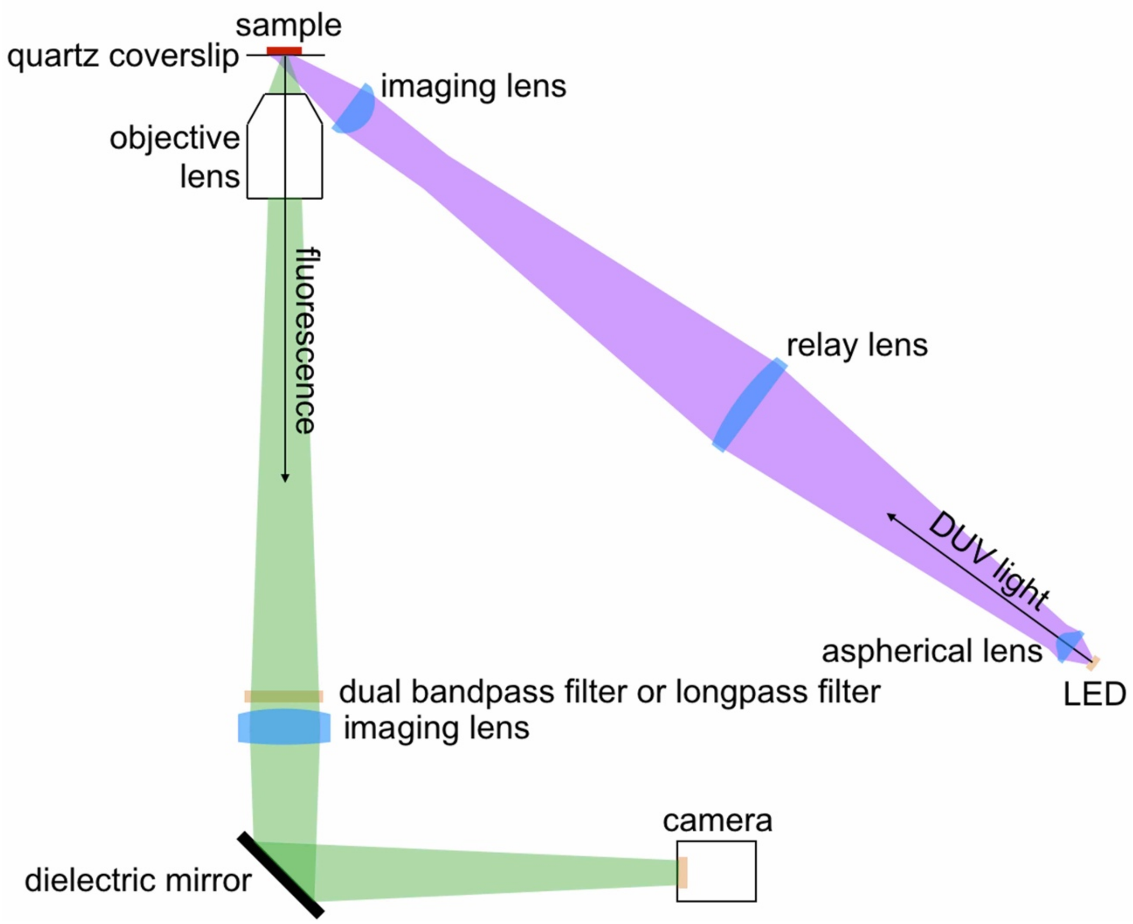

9. Fig. S7: A fluorescence hyperspectral imaging microscope at DUV excitation

We built a fluorescence hyperspectral imaging microscope at deep-UV excitation. A schematic view representing the microscope, drawn by Y.K., is shown. Details are described in Methods.

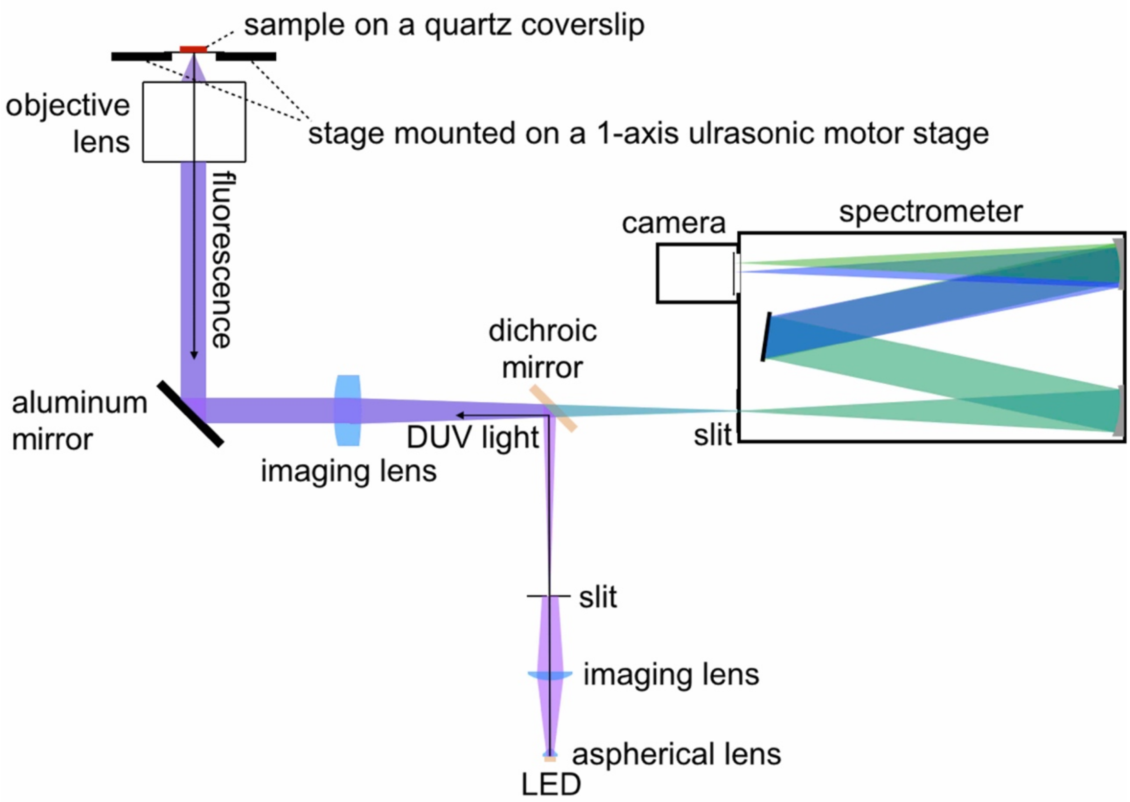
sample on a quartz coverslip
objective lens
stage mounted on a 1-axis ulrasonic motor stage
fluorescence
camera
spectrometer
dichroic mirror
aluminum mirror
DUV light
imaging lens
slit
slit
imaging lens
aspherical lens
LED
